# Unlocking the Potential of Caribbean Coarse Aggregates for High-Strength Concrete Development

**DOI:** 10.3390/ma18112503

**Published:** 2025-05-26

**Authors:** Adriana Mattos-Rodríguez, Andrés Guzmán, Daniel Abudinen

**Affiliations:** 1 Department of Civil and Environmental Engineering, Universidad de la Costa, Barranquilla 080002, Colombia; amattos@cuc.edu.co (A.M.-R.); dabudine@cuc.edu.co (D.A.); 2 Department of Civil and Environmental Engineering, Universidad del Norte, Barranquilla 081007, Colombia

**Keywords:** high-strength concrete, coarse aggregates, compressive strength, modulus of elasticity, Poisson’s ratio, Caribbean region

## Abstract

High-strength concretes (HSCs) are becoming increasingly important in modern construction, due to their ability to withstand high loads and reduce the size of structural elements. This study focuses on designing HSC mixes using materials readily available in the Colombian Caribbean region. The research involved preparing mixes with varying water–cement ratios (0.21 to 0.28) and incorporating a superplasticizer additive to maintain workability. The study evaluated the compressive strength gain of these mixes at different ages (14, 21, and 28 days). The results demonstrate that the materials available in the region, including the coarse aggregates and cement type, are suitable for producing HSC mixes with compressive strengths ranging from 55 to 84 MPa. Notably, the marble granite aggregate exhibited the best performance, achieving the highest compressive strength (84 MPa) with a water–cement ratio of 0.23. This mix also displayed favorable mechanical properties, with a modulus of elasticity of 36,000 MPa and a Poisson’s ratio of 0.26. These findings provide valuable insights for the development and application of HSC in the Colombian Caribbean region.

## 1. Introduction

### 1.1. Purpose of the Work and Significance

High-strength concrete (HSC), characterized by its superior compressive strength exceeding that of conventional concrete (f’c > 55 MPa, for HSC [1]), has become increasingly important in modern construction. This enhanced strength allows for the design of slender structural elements, leading to more efficient material use and innovative architectural designs. While the fundamental principles of concrete mix design still apply [2], achieving HSC necessitates a deeper understanding of the material properties and their interactions. This includes the careful selection of high-quality aggregates, optimization of the water–cement ratio, and the incorporation of admixtures to enhance workability and performance. The development of HSC mixes requires a nuanced approach, considering factors such as the desired strength, durability, and constructability. This research delves into the specific challenges and opportunities associated with developing HSC using materials available in the Caribbean region, contributing to the broader understanding of HSC design and application.

### 1.2. Background

#### 1.2.1. High-Strength Concrete Mixes

In the late 1960s, additives were first used in concrete mixes to reduce the use of water in material production (later known as superplasticizers). These advances were developed principally in Japan [3] and Germany [4]. The possibility of obtaining high-strength concretes with an important level of workability was noted as an ideal scenario for the construction of skyscrapers and large engineering works that require high-load strength in reduced spaces [2]. From that moment, developments related to new concrete technologies oriented toward the design of high-strength concrete mixes for military use have made it possible to obtain concrete with a strength higher than 400 MPa [1,5]. High-strength concrete is increasingly used in structural elements requiring reduced member size, increased load capacity, and improved durability. In some developed countries, compressive strengths above 100 MPa, reaching up to 140 MPa in special applications, have been reported [1].

High-strength concrete mixes involve the optimal use of normal concrete constituents. A low water–cement ratio would require a high quantity of cement if the mix is prepared using conventional materials. However, the appropriate selection of chemical additives and minerals can reduce the quantity of cement required, and it helps to obtain high-strength concrete in a much more affordable manner [6]. The water content depends on the desired workability, shape, form, and gradation of aggregates in fresh concrete. High-range water-reducing admixtures (HRWRs) enable a reduction in water content, while maintaining workability, thereby enabling the design of mixes with lower water–cement (w/c) ratios without compromising the fresh-state performance [7,8,9]. Worldwide experiences show that such concretes should have water–cement ratios ranging from 0.19 to 0.35 [10].

In the pursuit of improving the mechanical performance and sustainability of high-strength concrete (HSC), recent studies have explored the use of natural pozzolanic materials, such as calcined diatomaceous earth (CDE), as partial cement replacements [11,12]. Due to their high silica content and pozzolanic activity, these materials have been shown to refine the microstructure of the cement matrix and enhance both the strength and durability of concrete. Hasan et al. [12] demonstrated that incorporating CDE into HSC mixtures significantly improved the compressive strength, especially when used in combination with polypropylene and glass fibers. Further investigations confirmed that CDE not only enhanced the early and long-term strength, but also improved the resistance to chemical attack and elevated temperatures, in ultra-high-performance concrete (UHPC) [13]. Additionally, the synergy between CDE and fiber reinforcement has been identified as an effective strategy for optimizing the mechanical behavior of UHPC under complex loading conditions [14]. These findings support the growing interest in regionally adaptable, sustainable cementitious systems and offer a valuable reference framework for the use of alternative materials in tropical and resource-constrained environments.

#### 1.2.2. Mechanical Properties of High-Strength Concretes (HSCs)

HSC structures require local materials to be characterized according to their influence on concrete attributes. Optimal HSC necessitates an understanding of the mixture components and their behavior in order to update national structural design codes.

According to the literature, when the compressive strength of concrete increases, some of its characteristics and properties differ from regular concrete, such as the modulus of elasticity, tensile strength, flexural strength, and Poisson’s ratio [15]. The established norms in structural design codes are based on tests (proven experiences) performed with regular concrete. Lineal extrapolations obtained from regular concrete design equations in the approach to cases using HSC could lead to inadequate estimations of the real properties of the material. According to Rashid et al. [15], different proposals are found in the literature to predict the mechanical properties of HSC, but most of these proposals are limited to concretes with a maximal strength of 84 MPa. Back in 1985, before these proposals were established, studies on the structural properties of high-strength concrete, with a maximal strength of up to 84 MPa, were conducted [16].

In the Caribbean region, shallow approaches to HSC mixes have only recently been explored [12,17]. No data or equations are available for the prediction of the mechanical characteristics of HSCs fabricated with materials from the region. Although recent studies have demonstrated that soft computing techniques can effectively predict the compressive strength of ultra-high-strength concrete, such models have not yet been validated using aggregates and cementitious materials that are typical of the Caribbean region, which limits their direct applicability in this context [18]. Herrera and Mercado [17] reported concrete mix designs with a strength of up to 97 MPa, but with low plasticity, making them unmanageable. Meanwhile, in the Andina region, high-performance concretes have been developed with strengths close to 84 MPa, using native materials from the region [19].

Worldwide, high-rise building requirements promote the development of high-strength products to design buildings that can bear high loads, with structural elements of moderate dimensions. Four (4) key parameters are considered in the production of high-strength concrete: (i) a low water–cement ratio, (ii) the use of superplasticizers to maintain workability, (iii) the use of micro-void filling minerals, which prevent micro-fissure propagation, and (iv) the presence of high-quality coarse aggregates (necessary for good performance of the matrix–aggregate conglomerate).

HSC development requires the identification of the following mechanical properties to improve its usage: unconfined compression strength, complete stress–strain curves, modulus of elasticity, Poisson’s ratio, tensile strength, and shear strength [20].

While experimental characterization remains fundamental for assessing the mechanical properties of high-strength concretes, it is important to acknowledge the growing role of computational modeling techniques in advancing this field (not included in this study). In addition to laboratory testing, recent trends in concrete research have increasingly embraced computational methods, such as the Finite Element Method (FEM) and Molecular Dynamics (MD) simulations, to predict material behavior at different scales. FEM has been extensively applied for the macroscopic modeling of structural performance, enabling the analysis of the stress distribution, cracking, and damage evolution in concrete elements. In parallel, MD simulations offer atomistic-level insights into hydration reactions, bond formation, crack propagation, and the mechanical response of cementitious composites.

Notable reviews and studies, including those by Barbhuiya and Das [21], Li et al. [22], and Cao et al. [23], have highlighted the critical role of MD simulations in bridging nanoscale phenomena with macroscale mechanical behavior. These approaches complement experimental investigations and open up promising paths to improve the design, durability, and predictive capabilities for the development of concrete structures, especially in high-performance applications.

## 2. Materials and Methods

**Figure 1:**
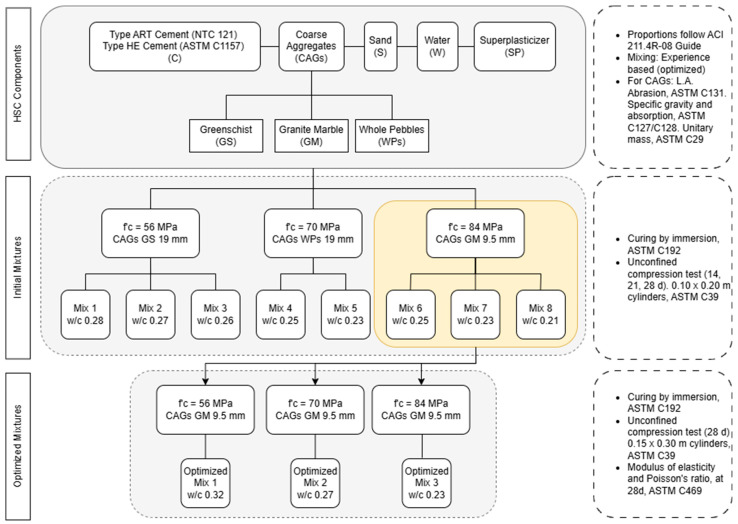
Flowchart summarizing the materials, mix designs, curing procedures, and testing program for the production and evaluation of high-strength concretes, following NTC 121 [24], ASTM C1157 [25], ASTM C131 [26], ACI 211.4R-08 Guide [27], ASTM C192 [28], ASTM C39 [29], ASTM C469 [30], ASTM C127 [31], ASTM C128 [32], and ASTM C29 [33].

The overall methodology followed in this study is summarized in Figure 1, detailing the materials used, mix designs, curing procedures, and testing program.

### 2.1. Material Selection

#### 2.1.1. Cement (C)

The cement used in this study was a Portland cement for structural use, commercially known as “*Cemento de Uso Estructural*”, produced by Argos, Colombia. It complies with the Colombian Technical Standard NTC 121:2021 [24], classified as Type ART (*Alta Resistencia Temprana*), which corresponds to Type HE (High Early Strength) under ASTM C1157-23 [25]. As a performance-based specification, the standard ensures that the cement achieves rapid early strength development, with a minimum compressive strength of 24 MPa at three days, without prescribing specific chemical compositions. This type of cement is particularly suitable for high-strength concrete applications that require fast strength gain, reduced permeability, and enhanced durability.

#### 2.1.2. Coarse Aggregates (CAGs)

The aggregates for this research were obtained entirely from the Caribbean region. Three types of coarse aggregates from different locations within this region were selected: grey shale, marble granite from Santa Marta, and whole pebbles (*canto rodado*) from Cartagena. The maximum nominal sizes of these aggregates ranged from 9.5 mm to 19 mm. Similar studies have been conducted in tropical marine environments using coral aggregates, highlighting the need for tailored concrete mix designs depending on local aggregate behavior and limitations [34]. The parameter considered to assess the adequacy of the aggregates was the resistance to degradation resulting from abrasion and impact using the Los Angeles test (as per ASTM C131 [26]). The higher the wear resistance parameter, the higher the compression strength of the rock [35]. Several tests, such as those measuring the specific gravity, absorption, and unitary mass, were performed to characterize the selected aggregates (Table 1).

In addition to the physical characterization of the CAGs, the following geological descriptions were considered to provide context on the origin and nature of the aggregates used.

Greenschist (GS) is a typical product made from the low-grade metamorphism of pelitic sediment rocks (rich in clay), derived from basic igneous rocks. In this particular case, GS involves a metamorphism with amphibolite facies [36].

Marble granite rock (GM) is mainly composed of quartz diorite, quartz monzonite, grain diorite, granite, and diorite. It has a phaneritic or coarse grain texture. Noting that the colors range from lighter to darker gray, it suggests that the mineralogical composition contains quartz diorite and diorite [37].

Meanwhile, whole pebbles (*canto rodado*, WPs) are sedimentary rock deposits formed of igneous rock fragments, metamorphic and other sedimentary rocks that acquire their rounded shape due to an erosion and transport process, causing their decomposition and disintegration. This study assesses the effect of water during the erosive process [38].

In addition to the characterization of the local aggregates used in this study, it is relevant to consider how their physical and mechanical properties compare to materials from other tropical and subtropical regions. Kabir et al. [39] reported that among granite, gneiss, and basalt aggregates used in Nigeria, basalt exhibited the highest compressive strength and bulk density, followed by gneiss and granite. The study reported Los Angeles abrasion values for these aggregates that ranged between 17% for basalt and up to 36% for granite, reflecting differences in the wear resistance of such aggregates. Similarly, González-Corominas et al. [40] evaluated natural and recycled aggregates in Spain, observing that crushed dolomite and river gravel presented a higher dry density and lower water absorption compared to recycled aggregates and ceramic materials, with abrasion losses below 30% for dolomite-based sources. These findings confirm that the mineral origin, porosity, angularity, and abrasion resistance significantly influence the compressive strength and durability of high-performance concretes. The marble granite aggregate used in the present study exhibited abrasion losses between 20% and 30%, placing it within the range of high-quality structural aggregates and suggesting its favorable performance in terms of mechanical behavior and durability.

#### 2.1.3. Fine Aggregate—Sand (S)

**Figure 2:**
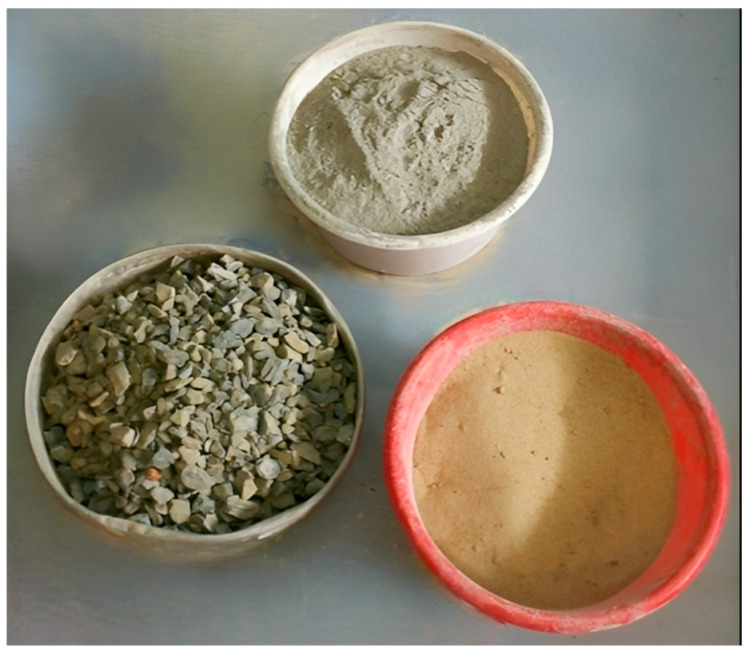
Coarse aggregate, fine aggregate, and cement prepared for conducting mix.

As a fine aggregate, this study contemplated using commercial sand from Santo Tomás (Atlántico) (Figure 2), mainly composed of silicon dioxide and minor quantities of quartz, mica, feldspar, and magnetite. The fineness modulus for the selected sand is 3.0, the specific gravity is 2.61, and the unit mass is 1.70 g/cm^3^.

Several studies have shown that fine aggregates with higher fineness modulus values tend to improve the mechanical performance and workability of high-strength concrete. Chang et al. [41], for instance, reported that coarser sands (FM ≈ 3.24) contributed to better compressive strength and reduced water demand compared to finer sands (FM ≈ 2.18), which increased the paste requirements and diminished the strength. Along the same lines, González-Corominas et al. [40] emphasized that sands with elevated absorption or ceramic content could negatively affect the strength development of cement. These findings support the adequacy of the selected fine aggregate in this study, which presented low absorption and a well-graded particle size distribution, favorable for the development of high-performance concretes.

#### 2.1.4. Additives

The selected additive was Sika Viscocrete 2100 (Sika, Barranquilla, Colombia) [42]. This liquid additive provides a high level of water (W) reduction and acts as a superplasticizer (SP). It is mainly composed of modified polycarboxylates. During the preliminary phase, different dosages were tested, concluding that the best results came about using the maximum recommended dosage suggested by the producer.

Silica fume was not included in the final mix designs. Preliminary tests incorporating this material showed compressive strength increases of less than 1%, which was not considered significant enough to justify its use for the purposes of this study.

### 2.2. Mix Design and Production

The proportions used to produce HSC were based on the recommendations in the ACI’s 211.4R-08 guide [27]. Eight mix designs were initially developed, targeting compressive strengths of 56 MPa, 70 MPa, and 84 MPa. The procedure followed the standard steps for selecting the water–cement ratio, adjusting the water content based on the fine aggregate void percentage, determining the cement content, and calculating the volumes of each component to achieve 1 m^3^ of concrete. A total air content of 2% was assumed in the mix design, corresponding to the estimated entrapped air in non-air-entrained high-strength concrete, in accordance with the ACI guidelines. Table 2 summarizes the proportions for the initial series of mixes. The final quantities were normalized to 1 m^3^ of concrete, following ACI 211.4R-08 recommendations.

In addition to the initial series of eight mixes, a second group of concrete designs was developed using the coarse aggregate that showed the best mechanical performance: marble granite (GM). These new mixes were designed to achieve compressive strengths of 56 MPa, 70 MPa, and 84 MPa, and followed the same proportioning methodology as described previously. The mixes were adjusted to achieve a target slump between 75 and 100 mm to improve workability. The detailed composition of these GM-based high-strength concretes is presented in Table 3.

The concrete batches were produced in a low-capacity mixer (~110 L), suitable for research-scale testing. For each of the eight initial mix designs, nine cylindrical specimens (100 mm × 200 mm) were prepared, with testing performed at 14, 21, and 28 days. Similarly, for the GM-based mixes, four cylindrical specimens (150 mm × 300 mm) per design were produced and cured for 28 days, prior to mechanical testing.

#### Mixing Procedure

Due to the characteristics of the mixes and materials used, such as low w/c ratios, angular coarse aggregates, and high-range water reducers, the mixing procedure was adapted to ensure proper workability and homogeneous dispersion of all the components. The process began with the pre-mixing of water and the superplasticizer. Approximately 40% of the mixing water was first introduced into the mixer and blended for 2 min. Cement was then added and mixed for 3 min, with the mixer paused intermittently to scrape off any cement adhered to the walls.

Subsequently, the aggregates were added simultaneously; first, the fine aggregate and then the coarse aggregate, and they were mixed for 10 min, while gradually incorporating the remaining 60% of the water during this stage. This extended mixing period was necessary to compensate for the low water content and ensure full dispersion. The adopted procedure minimized the risk of material sticking to the walls and ensured better paste–aggregate interactions under low workability conditions. This method represents a variation of a previous approach reported by Herrera and Mercado [17].

### 2.3. Production and Testing of Sample Cylinders

**Figure 3:**
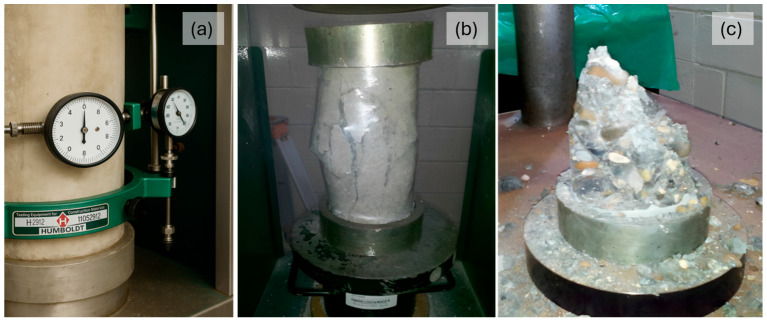
(**a**) Compressometer–extensometer installation for elastic property measurement; (**b**) plastic wrap protection; and (**c**) explosive failure of a 70 MPa design strength cylinder.

**Figure 4:**
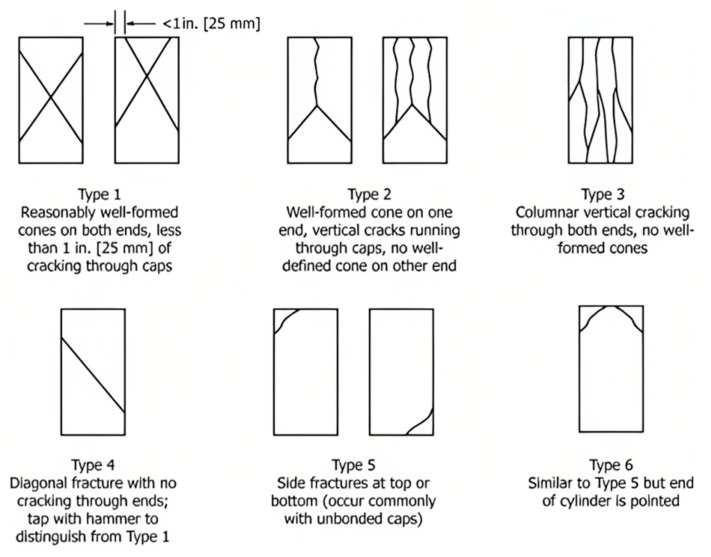
Failure types (FTs) in concrete cylinders [29].

For each mix, nine cylinders, measuring 0.10 × 0.20 m (4 × 8 in), were produced, which were removed from the mold 24 h after pouring and then cured [28]. After curing, the cylinders were dried, weighed, and then measured. Following the determination of the elastic properties (modulus of elasticity and Poisson’s ratio), the cylinders were covered with plastic wrap to prevent concrete fragments from flying off after compressive failure occurred (i.e., explosive failure) (Figure 3). The failure type was observed and classified using this procedure (Figure 4). The cylinders were tested at 14, 21, and 28 curing days, three cylinders for each age, which resulted in nine for each mix. The tests were performed using an automatic compressive strength machine (CONTROLS Automax 5 with 2000 kN capacity; CONTROLS S.p.A., Milan, Italy), applying a continuous loading rate of 0.25 MPa/s, in accordance with ASTM C39/C39M-24 specifications [29].

The variability in compressive strength measurements was analyzed using basic statistical tools. For each concrete mix and curing age, the mean and standard deviation were calculated based on three independent specimens. The standard deviation was used to quantify the dispersion of the data, and boxplots were constructed to visually represent the distribution, including the interquartile range (IQR), median, and data range. This statistical treatment follows the methodologies recommended for engineering experimental data analysis [44].

### 2.4. Modulus of Elasticity and Poisson’s Ratio Testing

The static modulus of elasticity and Poisson’s ratio were determined according to ASTM C469/C469M-22 [30]. The test was conducted on cylindrical concrete specimens after 28 days of curing. For the initial eight concrete mixes, three specimens per mix were tested, using 100 mm × 200 mm cylinders. In the case of the optimized mixes using granite marble (GM), four specimens per mix were prepared and tested, using 150 mm × 300 mm cylinders.

The specimens were removed from the curing pool, and their diameter, height, and weight were recorded within one hour, enabling the calculation of the concrete density for a later comparison with the model-predicted elastic modulus values.

A compressometer–extensometer assembly connected to a data acquisition system was used to measure the longitudinal and transverse deformations. The compressive load applied was limited to 40% of the average compressive strength of the corresponding concrete type, as specified by the relevant standards. These measurements enabled the identification of the linear portion of the stress–strain curve, from which both the modulus of elasticity and Poisson’s ratio were calculated.

## 3. Results and Discussion

### 3.1. Workability Observations

The fresh workability of the concrete mixes varied depending on the aggregate type and water–cement ratio. Mixes 1, 2, 4, 5, and 6 exhibited good workability, enabling easy pouring and proper vibration without complications. In contrast, mixes 3, 7, and 8 showed medium workability, which led to difficulties during casting and compaction. These observations are consistent with the expected influence of low water content and aggregate texture on the rheological behavior of high-strength concrete.

### 3.2. Failure Type

**Figure 5:**
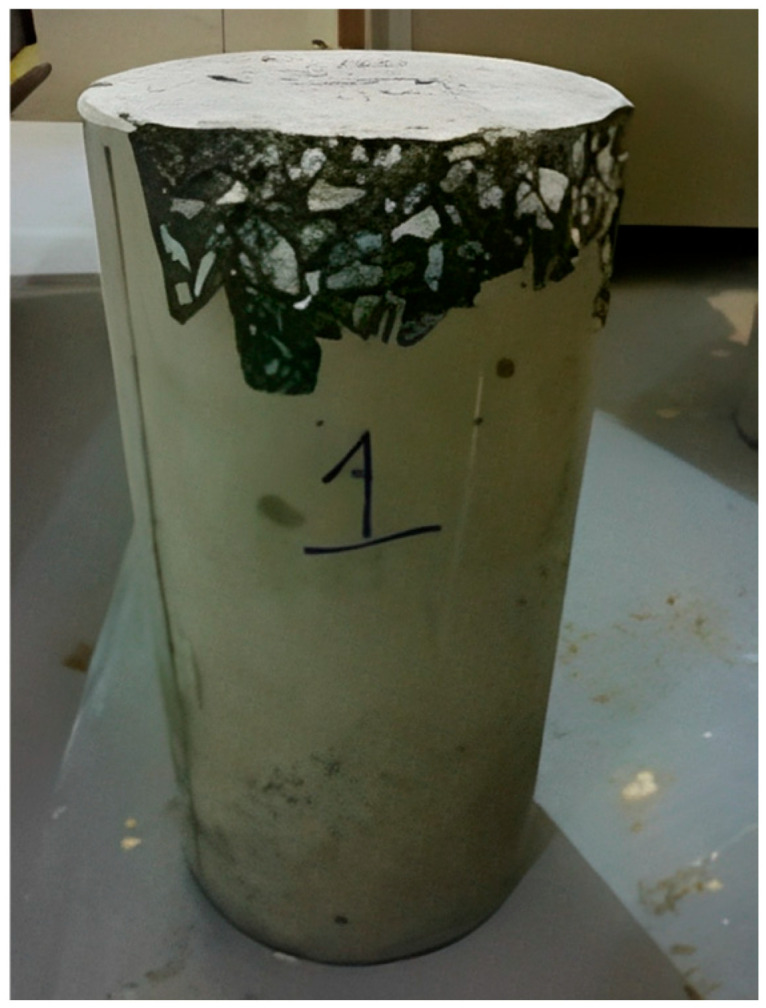
Failure type 5—mix 6.

The failure type enables observations to be made regarding the testing method and specimen quality, considering the capping or specimen placed inside the test machine (Figure 4). The unconfined compressive strength results for the cylinders at different ages (following ACI 363.2 R11 guide, [45]) and the failure type for each mix design are presented in Table 4. The failure type of the cylinders tested at 14 days (type 5) indicates that the headers were not properly adhered (Figure 5). In the posterior tests, neoprene pads with SHORE 70 hardness were used, because during testing columnar and diagonal failure types were observed.

At lower compressive strength levels (56 MPa mixes—mixes 1, 2, and 3), the predominant failure mode observed was type 3: vertical cracking through both ends without well-formed cones. These concretes were produced with greenschist coarse aggregates (nominal size 19 mm). The relatively higher porosity of the cement matrix, coupled with the metamorphic nature of greenschist (with moderate angularity and moderate abrasion resistance), led to progressive crack propagation along the specimen’s vertical axis. The moderate confinement provided by the aggregates and the moderate bonding at the matrix–aggregate interface favored the development of axial cracks rather than well-defined conical failures.

At intermediate strength levels (70 MPa mixes—mixes 4 and 5), the predominant failure mode was type 5: side fractures occurring at the top or bottom of the cylinders. These mixes used whole pebbles (nominal size 19 mm) as coarse aggregates. Whole pebbles are rounded sedimentary rocks with low surface roughness and moderate mechanical resistance, which limits the mechanical interlock and bond strength with the matrix. This contributed to localized stress concentrations and promoted side fractures near the cylinder ends.

At the highest strength levels (84 MPa mixes—mixes 6, 7, and 8), the predominant failure mode was type 4: diagonal fracture without cracking through the ends. These concretes incorporated marble granite aggregates (nominal size 9.5 mm), which are of igneous origin, and are highly angular and possess high abrasion resistance. The high density of the cement matrix, the strong mechanical interlock due to the angular aggregates, and the reduced maximum aggregate size favored an abrupt shear-dominated failure mechanism. In this case, the material’s capacity to dissipate energy through microcracking was minimized, leading to brittle diagonal fractures, indicative of high internal cohesion and matrix–aggregate bond strength.

### 3.3. Design vs. Experimental Comparison of Compressive Strength

**Figure 6:**
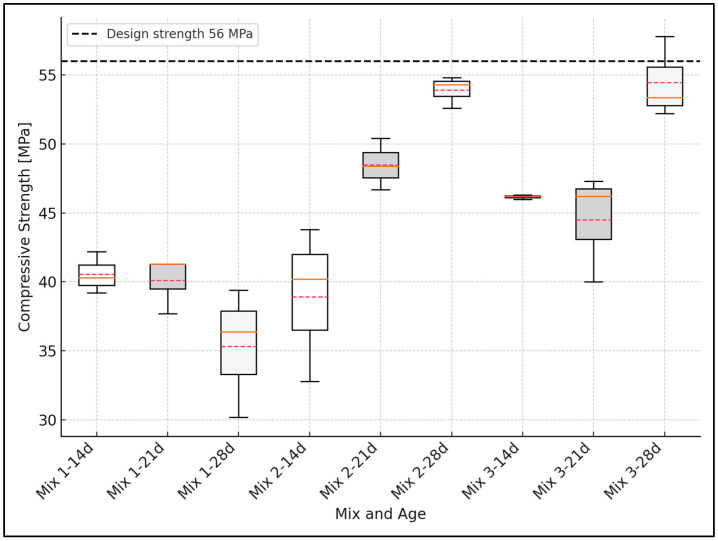
Compressive strength vs. concrete curing time (mixes 56 MPa).

**Figure 7:**
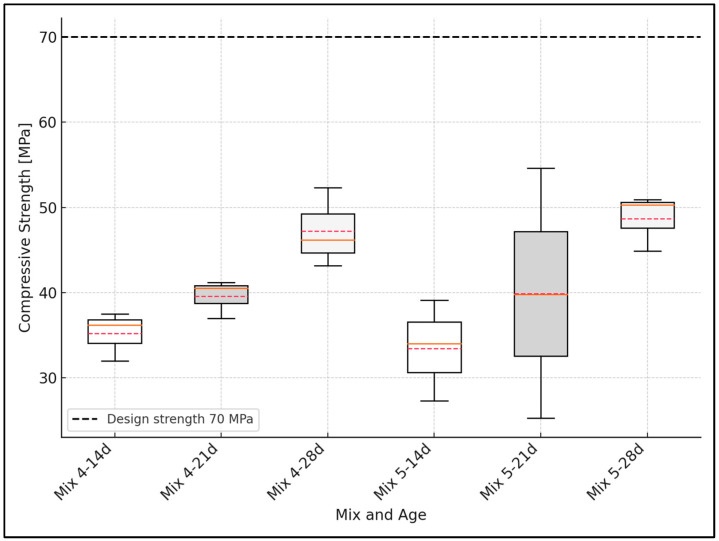
Compressive strength vs. concrete curing time (mixes 70 MPa).

**Figure 8:**
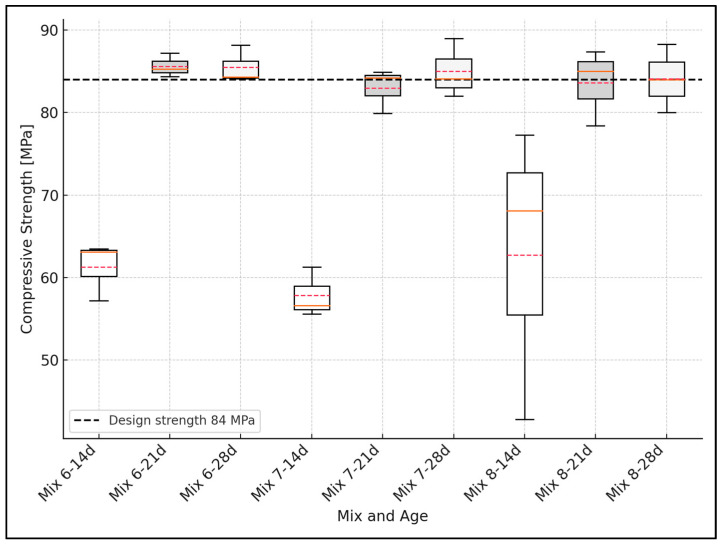
Compressive strength vs. concrete curing time (mixes 84 MPa).

Figure 6, Figure 7 and Figure 8 show the compressive strength development for concrete mixes at 14, 21, and 28 days. As stated in Section 2.3, the error analysis was based on the standard deviation calculated for each concrete mix and curing age. In the boxplot representations, the boxes correspond to the interquartile range (IQR), the whiskers represent the data range, the solid red line indicates the mean value, and the dashed red line marks the median value of the results. The black dashed line represents the target design strength.

For 56 MPa mixes made using greenschist (GS) aggregate, the first water–cement ratio of 0.28 reached 64% of the designed strength, in contrast to mixes 2 and 3, which reached 97.7 and 98.7% (Figure 6).

Mixes 4 and 5, designed for compressive strengths of 70 MPa, failed to develop the expected strength. These mixes with whole pebble (WP) aggregate reached 68.5 and 70.6% of the design strength, respectively (Figure 7).

Finally, the 84 MPa mixes, 6, 7, and 8, reached the design strength and exceeded it. These mixes made with marble granite (GM) reached 103.4, 102.8, and 101.6% of the design strength, respectively (Figure 8). However, despite this, these mixes have low workability, which complicates their manufacture and their use in practice. This situation could be improved by increasing the maximum recommended dose of superplasticizer.

Given the results obtained in the compression tests performed on the different mixes designed, the significant influence of the aggregate type and its characteristics on the strengths achieved are noteworthy. The effect of aggregate density is not notable for HSC, but the aggregate size and type, water–cement ratio, and concrete density have effects on the compressive strength of HSC [46].

In the case of GS aggregate, it was advisable to make use of a water–cement ratio of 0.40, according to Table 6.5 of ACI 211.4R-08 [27]. However, after developing five sample mix designs with water–cement ratios ranging from 0.28 to 0.35, it was noticed that the expected strength was reached with the lowest ratio. For this reason, the new mixes prepared had water–cement ratios ranging from 0.26 to 0.28. As a result, mixes 2 and 3 had improved strength properties, reaching more than 95% of the design strength. Notably, this kind of aggregate had an angular shape and a high percentage of fractured faces, which guaranteed the desirable adherence between the aggregate and the mix.

Furthermore, the mixes with a design strength of 70 MPa did not reach 85% of the said strength at 28 days. The aggregate used had a rounded shape, a smooth texture, and a diameter of 19 mm. At the time of the failure of the specimens, the aggregate seemed segregated into the mix; it could become detached easily, demonstrating the poor adhesion between the aggregate and the mix. Minimal segregation, among other mix characteristics, is essential to achieve high strength in concrete [47].

The aggregate used in the 84 MPa mixture had an angular shape, with diameters that were not over 9.5 mm. The good adhesion between the aggregate and the matrix was evident in these concrete cylinders. Despite honeycombing in some specimens caused by the use of inappropriate vibration techniques, the expected design strength remained. The strength obtained even exceeded the design strength (Figure 8).

### 3.4. Relationship Between the Compressive Strength and the Water–Cement Ratio

The strength of structural concrete is controlled by the strength of its hardened cement paste [48], which is related to its w/c ratio. The authors state that the microporosity of the cement paste in the concrete is a direct function of the w/c ratio; thus, the higher the w/c ratio, the higher the porosity in the paste, and, consequently, the weaker the concrete.

**Figure 9:**
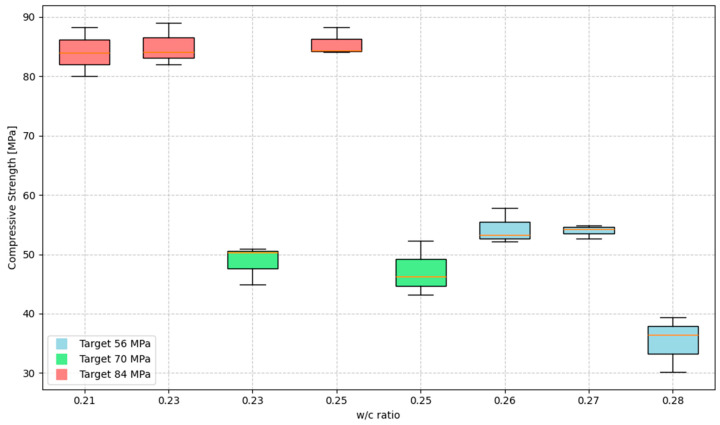
Compressive strength vs. w/c ratio.

The 56 MPa mixes were designed with w/c ratios of 0.26, 0.27, and 0.28. As shown in Figure 9, the compressive strength generally increased with a decreasing w/c ratio. However, the strength measured for mix 1 (w/c = 0.28) was significantly lower than expected. This drop may be attributed to the concrete’s poor initial workability, limited consolidation, and the use of neoprene pads that did not perform adequately during testing. These factors likely affected the load transfer and induced early failure, rather than reflecting a true material deficiency.

The 70 MPa mixes had water–cement ratios of 0.23 and 0.25, lower than the 56 MPa mixes; nevertheless, they did not reach 85% of the design strength, mainly due to the lack of coarse aggregate adherence to the cement paste (Figure 3).

In contrast to the previously discussed mixes, the 84 MPa mixes showed the opposite behavior, since the strength remained with respect to the curing time, instead of decreasing with the increasing water–cement ratio. It is possible to argue that the strength remained almost constant for all the water–cement ratios considered in this design (0.21, 0.23, and 0.25). Likewise, it is outstanding that despite the strength of the other mixes that increased as the water–cement ratio decreased, these mixes also showed poor workability and, therefore, the presence of honeycomb or coarse aggregate segregation in most of the tested cylinders. In conclusion, the result is a consequence of using improper vibration procedures, due to the low workability of the cement because of its low water–cement ratio.

### 3.5. Aggregate Size vs. Compressive Strength

**Figure 10:**
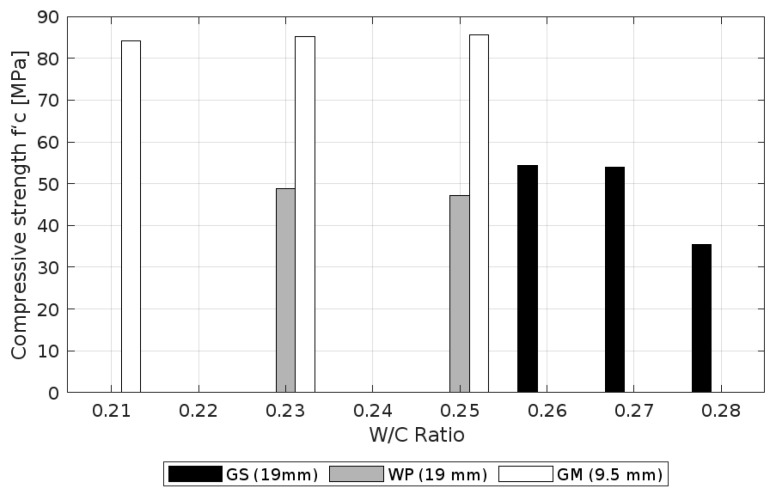
Compressive strength vs. w/c ratio, highlighting the coarse aggregate influence.

It is important to clarify and remember the sizes of the three aggregates used for this research, namely two that had a diameter of 19 mm and the other with a diameter of 9.5 mm, despite the recommendations in ACI 211.4R-08 [27], which suggests using aggregates with diameters of less than 12.7 mm for the development of high-strength concrete. The reason for this is because one of the main objectives of this work is to use aggregates that are available on the market. The use of smaller aggregates contributed to achieving higher strength mixes with similar water–cement ratios, as evidenced in Figure 10. Likewise, the mixes with greater strength contained an aggregate with a diameter of 9.5 mm, appropriate for the development of HSC mixes; an aggregate with a diameter of 7 mm or below is suitable for Very-High-Strength Concrete [46]. The same aggregate was subsequently selected to for use in three mix designs, with the strength initially established and in combination with the use of a filling mineral.

### 3.6. Modulus of Elasticity and Poisson’s Ratio

To evaluate the validity of existing empirical models in predicting the modulus of elasticity for high-strength concrete (HSC), a comparative analysis was carried out using several international formulations. Table 5 summarizes the selected expressions from the relevant codes and the literature, including ACI, AASHTO-LRFD, CEB-FIP, and others.

The experimental results obtained for the different mix designs are presented in Table 6. As expected, the mixes designed with lower water–cement ratios generally exhibited higher elastic modulus values. For the 84 MPa mix (w/c = 0.23), the modulus of elasticity averaged 36,212 MPa, while for the 70 MPa and 56 MPa mixes (w/c = 0.27 and 0.32), the values decreased accordingly. These findings are consistent with the expected influence of matrix density and microstructure refinement on stiffness development.

Moreover, although not the focus of the modulus testing, the GM-based mixes also exhibited improved fresh workability, likely due to the optimized slump range and reduced maximum aggregate size.

Poisson’s ratio values ranged between 0.22 and 0.28 across the tested mixes. A slight increase in Poisson’s ratio was observed as the water–cement ratio decreased, which may be attributed to better matrix compaction and improved aggregate–matrix interface bonding. These values slightly exceed the typical range of 0.15–0.22 reported for conventional concretes in the elastic domain [2,49], which is reasonable considering the dense microstructure developed in high-strength concretes. It is important to note that Poisson’s ratio can increase significantly near ultimate failure due to internal microcracking and lateral dilatation; however, the values reported in this study were determined within the elastic range. Additional tests would be needed to further confirm this trend and its behavior under different loading conditions.

When compared to international prediction models, the experimental results for the modulus of elasticity aligned most closely with the ACI (1992) and Norges (1992) formulations, showing differences below 5%. Conversely, empirical models, such as AASHTO-LRFD and Eurocode 2, tended to overestimate the modulus by up to 17%. These discrepancies underscore the importance of validating international models with region-specific materials, as the aggregate type and microstructure significantly affect the stiffness of high-strength concrete.

The most notable discrepancies were observed in the comparisons with the AASHTO, Eurocode 2, and Iravani formulations, with deviations reaching up to 25% (Table 6). These differences are consistent with the findings reported by Vakhshouri and Nejadi [50], who emphasized the strong influence of the aggregate type and characteristics on the mechanical behavior of HSC. Since aggregate types vary significantly across regions, the materials used in this study were inherently different from those assumed in the development of the referenced empirical models. In contrast, the equations proposed by ACI, Ahmad and Shah, and Norges, exhibited better agreement with the experimental results, with deviations below 11%.

**Figure 11:**
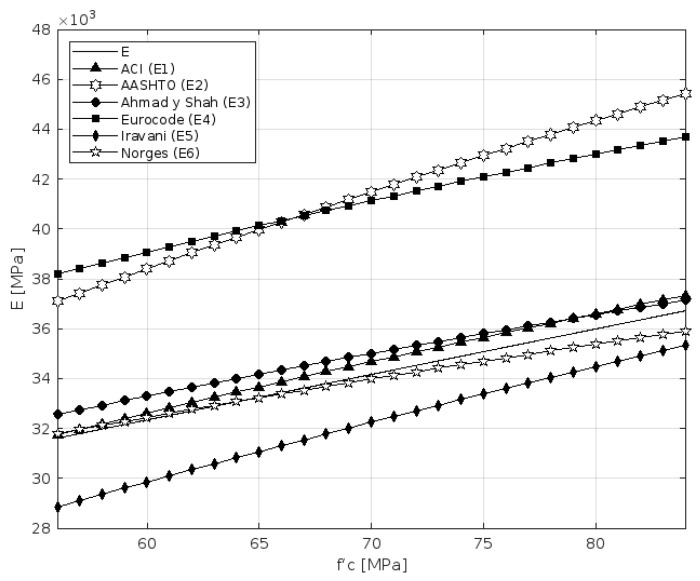
The relationship between the modulus of elasticity and compressive strength.

A linear adjustment was computed for the elastic modulus obtained in the tests performed (Figure 11). The results support the analysis with respect to the comparison made with the expressions established in Table 5; an average concrete specific weight (avg. wc) of 2369 was used in the AASHTO equation. The dotted line shows that the adjustment is nearest to the equations by Norges and ACI, but not so far from the Ahmad and Shah equation, as previously mentioned.

The mixes had water–cement ratios of 0.32, 0.27, and 0.23 for the 56, 70, and 84 MPa designs, respectively. For these mixes, the range of values reported in this study for Poisson’s ratio enables the increase to be checked by decreasing the water–cement ratio (Table 6).

Despite the linear relationship in regard to the trend line between f’c^(1/2)^ and the estimated value of E, standardizing the abscissa to f’c^(1/3)^ yields an R^2^ value closer to one (R^2^ = 0.9998), as per Equations (1) and (2).

E = 4089.7 × f’c^(1/2)^ (1)

E = 8316.7 × f’c^(1/3)^ (2)

A recent case study in Colombia focused solely on one concrete supplier and reported on the effect of the aggregates used on the elastic modulus for normal and high-strength concretes [51]. This concrete supplier has the ability to select and blend aggregates from across the country to improve mix performance. The study presented a regional context for the production of high-strength concrete in the Caribbean. Both studies could be considered in the near future to evaluate potential results in regard to the best approximations for concrete mix design optimization.

Recent studies have explored the use of alternative materials, such as calcined diatomaceous earth, as supplementary cementitious components in high-strength concrete, particularly in tropical environments. While the aggregates evaluated in this study, greenschist, marble granite, and whole pebbles, serve primarily as the structural skeleton of the concrete matrix, calcined diatomaceous earth acts as a fine pozzolanic additive that enhances the microstructure and long-term durability of concrete. Although not commonly found in the Caribbean region, the comparison underscores a broader trend in adapting concrete formulations to locally available resources. Unlike the coarse aggregates used in this work, which exhibit high density and rough textures, calcined diatomaceous earth is characterized by its low density and high porosity. These differences highlight complementary approaches: one based on optimizing aggregate selection for mechanical performance, and another based on chemical reactivity and microstructural refinement. Future studies could consider hybrid solutions that combine local aggregates with supplementary materials to further enhance concrete performance, particularly under the constraints of tropical climates.

## 4. Conclusions

One of the main purposes of this research was to analyze the effects of the water–cement ratio, the influence of the type and size of aggregates from the Caribbean region, and the dosages of materials in developing high-strength concretes. The results on the unconfined compressive strength of the developed concretes showed an influence on the strength reported due to the size, shape, and texture of the aggregate used. Likewise, it was observed that lower water–cement ratios, in conjunction with the aggregates with diameters smaller than 1/2 inch (12.7 mm), influence the achievement of concrete strengths equal to or greater than 70 MPa.

On the other hand, the aggregates’ shape and texture are important variables due to their direct influence on the binding between the matrix (pasta) and the aggregate. If good adhesion is not achieved, it contributes to the failure between these connections and could also cause the failure of the specimen in regard to the attainment of a strength lower than designed. It is recommended that aggregates with a high percentage of fractured faces and rough textures are used to ensure adherence with the matrix.

For mixes with a design strength of 56 MPa, failure was usually observed in the aggregate (the shale aggregate presents a foliated texture, and it can be easily broken into sheets, which generate fault planes), and occurred in regard to the decapitation of the same specimen, a consequence of the non-adherence among older neoprene pads. In the 70 MPa mixes, failure occurred in the paste or in the matrix–aggregate binding as a product of poor adhesion between them. Finally, in the 84 MPa mixes, the failure occurred throughout the conglomerate, both in the paste and the aggregate.

Superplasticizers were added to some of the concrete mixes to improve their workability. However, in some cases (mixes 5, 7, 8), this effect was not altogether outstanding due to the low water–cement ratio used in their design. Hence, establishing the appropriate water–cement ratio is recommended not only to improve the strength of concrete, but also in order to increase the proportion of superplasticizers used.

The experimental modulus of elasticity in the final mix designs, developed with marble granite, were more consistent with the equations proposed by ACI (1992) and Norges (1992), both with an average difference percentage of 3,5. The AASTHO and Eurocode (1993) equations overestimate the modulus of elasticity by an average of 17%. The comparison with the ACI, Norges, and Ahmad and Shah equations, showed very low percentage differences, which validates and accredits the measurements used in our study. Similarly, the calculated Poisson’s ratio values are within the normal ranges set forth in the literature on high-strength concrete. An increase in the variability in this ratio was also observed by decreasing the water–cement ratio.

The final mix designs incorporated marble granite with a diameter of 9.5 mm as the coarse aggregate, with a 10% cement replacement using a filler material. Although the inclusion of this filler contributed to early-age strength development, it also increased the mix density and reduced its workability. To address this, the superplasticizer dosage was increased relative to the water content, and the mix design was adjusted to achieve higher slump values. Based on these observations, future designs should prioritize angular, rough-textured aggregates, with nominal sizes equal to or below 12.7 mm, and consider the combined use of plasticizers and superplasticizers to maintain adequate workability in low w/c ratio conditions.

Although the findings of this study provide valuable insights into the mechanical performance of high-strength concrete produced with regionally available aggregates in the Caribbean, some limitations must be acknowledged. The experimental program focused primarily on the compressive strength, elastic modulus, and Poisson’s ratio of concrete, without including other important aspects, such as tensile strength, shrinkage, creep, or durability. In particular, the long-term performance of concrete under tropical environmental conditions, such as high humidity, temperature variations, and aggressive exposure, remains an open research question. Future studies should address these durability-related aspects through comprehensive testing protocols and possibly explore hybrid mix designs, incorporating supplementary cementitious materials. Additionally, expanding the number of mixtures and incorporating statistical experimental designs could help further validate the influence of aggregate characteristics on high-strength concrete behavior in tropical regions.

## Figures and Tables

**Table 1:** Physical and mechanical properties of coarse aggregates.

Type of CAGs	Abrasion Test (%) L.A. (ASTM C131)	Specific Gravity	Absorption (%)	Unitary Mass [g/cm^3^]	Aggregate Size [mm]
Greenschist (GS)	25.73	2.90	1.83	1.60	19.0
Granite marble (GM)	22.78	2.68	2.59	1.51	9.5
Whole pebbles (WPs)	23.04	2.63	2.04	1.77	19.0

**Table 2:** Mix designs and proportions to produce 1 m^3^ of concrete.

Mix Design	1	2	3	4	5	6	7	8
f’cr (28 days) [MPa]	56			70		84		
CAG diameter [mm]	GS, 19			WP, 19		GM, 9.5		
Fineness modulus	3			3		3		
Volume unity CAGs ^1^	0.72			0.72		0.65		
CAG weight [kg]	1152			1274		983.45		
W content [L] ^2^	175.0			175.0		189.8		
Slump [mm] [43]	50–75			50–75		50–75		
w/c ratio ^3^	0.28	0.27	0.26	0.25	0.23	0.25	0.23	0.21
Wcm [kg]	616.27	639.10	663.68	690.22	750.24	749.50	814.68	892.27
Ws [kg]	559.10	540.19	519.82	269.92	220.19	488.99	434.99	370.70
SP [%]	0.8			0.8		0.8		
Wsp [kg]	4.93	5.11	5.31	1.77	19.0	6.00	6.52	7.14

^1^ ACI 211.4R, Table 4.3.3; ^2^ ACI 211.4R, Table 4.3.4; ^3^ ACI 211.4R, Table 4.3.5(b).

**Table 3:** Marble granite mix design to produce 1 m^3^ of concrete.

Mix Design	1	2	3
f’cr (28 days) [MPa]	56	70	84
CAG diameter [mm]	GM, 9.5		
Volume Unity CAG ^1^	0.65		
CAG weight [kg]	983.45		
W content [L] ^2^	189.7		
w/c ratio ^3^	0.32	0.27	0.23
Wcm [kg]	585.11	693.47	814.07
Ws [kg]	625.56	535.78	435.86
SP [%]	1.60		
Wsp [kg]	9.36	11.10	13.03

^1^ ACI 211.4R, Table 4.3.3; ^2^ ACI 211.4R, Table 4.3.4; ^3^ ACI 211.4R, Table 4.3.5(b).

**Table 4:** Compression tests results and representative failure type [29].

Mix Design	f’c 14 d [MPa]			Avg. f’c 14 d [MPa]	FT ^1^	f’c 21 d [MPa]			Avg. f’c 21 d [MPa]	FT	f’c 28 d [MPa]			Avg. f’c 28 d [MPa]	FT	Exp. f’c ^2^ 28 d [MPa]	%
1	39.2	40.3	42.2	40.6	5	41.3	37.7	41.3	40.1	5	39.4	30.2	36.4	35.3	5	55.2	64.1
2	32.8	43.8	40.2	38.9	5	46.7	50.4	48.4	48.5	4	52.6	54.8	54.3	53.9	3	55.2	97.7
3	46.3	46.2	46.0	46.2	5	47.3	46.2	40.0	44.5	5	53.4	52.2	57.8	54.4	3	55.2	98.7
4	32.0	37.5	36.2	35.2	5	41.2	37.0	40.5	39.6	5	43.2	52.3	46.2	47.2	5	68.9	68.5
5	27.3	39.1	34.0	33.5	5	39.8	25.3	54.6	39.9	5	50.9	44.9	50.3	48.7	5	68.9	70.6
6	63.5	57.2	63.1	61.3	5	84.4	85.3	87.2	85.6	3	88.2	84.3	84.1	85.6	4	82.7	103.4
7	55.6	56.6	61.3	57.8	5	84.2	79.9	84.9	83.0	4	89.0	84.1	82.0	85.1	4	82.7	102.8
8	42.8	77.3	68.1	62.7	5	85.0	87.4	78.4	83.6	3	84.0	80.0	88.3	84.1	4	82.7	101.6

^1^ FT: failure type; ^2^ Exp. f’c: expected (specified) strength.

**Table 5:** Expressions for the modulus of elasticity of concrete, Ec [15].

Standard/Researcher (Ref. for Table 6)	Ec Equation [MPa]	Compressive Strength Range
ACI (1992) [1]	3320 × (f’c)^0.5^ + 6900	21 MPa < f’c < 83 MPa
AASHTO-LRFD [2]	0.043 × (wc)^1.5^ × (f’c)^0.5^	Not specified
Ahmad and Shah (1985) [3]	8800 × (f’c)^0.325^	f’c < 84 MPa
CEB-FIP (1993) (EuroCode2) [4]	21,500 × _αE_ × (f’c/10)^1/3^	f’c < 80 MPa
Iravani (1996) [5]	4700C_ca_ × (f’c)^0.5^	55 MPa < f’c < 125 MPa
Norges (1992) [6]	9500 × (f’c)^0.3^	25 MPa < f’c < 85 MPa

**Table 6:** Experimental and calculated values for modulus of elasticity (E) and Poisson’s ratio (ν).

Sample		wc	ν	E	E(1)	%E(1)	E(2)	%E(2)	E(3)	%E(3)	E(4)	%E(4)	E(5)	%E(5)	E(6)	%E(6)
(84 MPa)	1	2398	0.27	36,212	37,328	3.0%	46,285	21.8%	37,143	2.5%	43,705	17.1%	35,322	2.5%	35,893	0.9%
	2	2378	0.28	37,479	37,328	0.4%	45,715	18.0%	37,143	0.9%	43,705	14.2%	35,322	6.1%	35,893	4.4%
	3	2438	0.26	36,131	37,328	3.2%	47,448	23.9%	37,143	2.7%	43,705	17.3%	35,322	2.3%	35,893	0.7%
	4	2359	0.28	38,022	37,328	1.9%	45,141	15.8%	37,143	2.4%	43,705	13.0%	35,322	7.6%	35,893	5.9%
(70 MPa)	5	2376	0.28	33,763	34,677	2.6%	45,647	26.0%	35,006	3.5%	41,128	17.9%	32,245	4.7%	33,982	0.6%
	6	2399	0.28	32,677	34,677	5.8%	42,272	22.7%	35,006	6.7%	41,128	20.5%	32,245	1.3%	33,982	3.8%
	7	2337	0.27	33,465	34,677	3.5%	40,653	17.7%	35,006	4.4%	41,128	18.6%	32,245	3.8%	33,982	1.5%
	8	2351	0.27	34,777	34,677	0.3%	41,015	15.2%	35,006	0.7%	41,128	15.4%	32,245	7.9%	33,982	2.3%
(56 MPa)	9	2307	0.27	29,103	31,745	8.3%	35,653	18.4%	32,557	10.6%	38,180	23.8%	28,841	0.9%	31,782	8.4%
	10	2385	0.22	34,279	31,745	8.0%	37,473	8.5%	32,557	5.3%	38,180	10.2%	28,841	18.9%	31,782	7.9%
	11	2335	0.23	32,854	31,745	3.5%	36,309	9.5%	32,557	0.9%	38,180	13.9%	28,841	13.9%	31,782	3.4%
	12	2360	0.24	31,183	31,745	1.8%	36,881	15.5%	32,557	4.2%	38,180	18.3%	28,841	8.1%	31,782	1.9%
avg. wc		2369														

## Data Availability

The raw data supporting the conclusions in this article will be made available by the authors upon request.

## Abbreviations

The following abbreviations are used in this manuscript:

CAGs	Coarse aggregates
Dw	Water density
E	(Tangent) Modulus of elasticity
FTs	Failure types
GM	Granite marble
GS	Greenschist
HE	High-early strength (cement type HE)
HSCs	High-strength concretes
HRWRs	High-range water-reducing admixtures
IQR	Interquartile range
S	Sand
S1	Stress corresponding to strain ε1
S2	Stress corresponding to 40% of the ultimate load for concrete
SGx	Specific gravity
SP	Superplasticizer
V	Void percentage in sand
Vair	Air volume
Vcag	Coarse aggregate volume
Vcm	Cement volume
Vfa	Fine aggregate volume
Vw	Water volume
Vx	Mix volume for each constituent material
W	Water
wc	Concrete specific weight
w/c	Water–cement ratio
Wcm	Cement weight
Wfa	Fine aggregate weight
WPs	Whole pebbles
Ws	Sand weight
Wsp	Superplasticizer weight
Ww	Water weight
Wx	Mix weight for each constituent material
ε2	Longitudinal strain produced in S2
εt1	Transverse strain according to the average height of the specimen, produced by stress S1
εt2	Transverse strain according to the average height of the specimen, produced by stress S2
f’c	Compressive strength of concrete
μ	Poisson’s ratio
